# Von Ziemssen’s Cyclopedia

**Published:** 1875-01

**Authors:** 


### Bibliographical.

CYCLOPEDIA OF THE PRACTICE OF MEDICINE. Edited by Dr. H. Von Ziemssen, Professor of Clinical Medicine in Munich, Bavaria. New York: Wm. Wood & Co.; 1874.

The first volume of this valuable series has been received, but not in time for revision and such notice in this number of the JOURNAL as it deserves.

For years physicians have felt the great need of a work which should fully correspond to the present standpoint of clinical medicine, embracing, as this work does, the entire range of special pathology and therapeutics. The ordinary text-books which are offered to the profession in such profusion do not supply this want. The object of the *Cyclopedia* is to furnish a "complete picture of the present state of medical knowledge in the departments of etiology, pathology, and treatment," and from the volume before us, we feel assured that the object will be fully accomplished.

In our next issue, we propose to notice some of the prominent features of this great work, which we feel may be regarded as the richest contribution to medical literature during the past half century.

